# MAFLD/MASLD: Past, Present, and Future

**DOI:** 10.1111/jgh.70619

**Published:** 2026-08-11

**Authors:** Vincent Wai‐Sun Wong

**Affiliations:** ^1^ Medical Data Analytics Centre, Department of Medicine and Therapeutics The Chinese University of Hong Kong Hong Kong China; ^2^ State Key Laboratory of Digestive Disease, Institute of Digestive Disease The Chinese University of Hong Kong Hong Kong China

**Keywords:** diabetes, fatty liver, liver fibrosis, obesity, steatotic liver disease

## Abstract

Metabolic dysfunction‐associated steatotic liver disease (MASLD), also known as nonalcoholic fatty liver disease and metabolic dysfunction‐associated fatty liver disease, affects more than 30% of the global adult population and has become one of the leading causes of cirrhosis and hepatocellular carcinoma. From what we are seeing today, it is hard to imagine that only 25 years ago the disease was considered unimportant, liver biopsy was the only means of assessing disease severity, and no pharmaceutical company was developing treatments for steatohepatitis (MASH). In this Okuda State‐of‐the‐Art Lecture, I discuss the evolution of our understanding of MASLD over the past 25 years and highlight the development of noninvasive biomarkers and clinical care pathways. The accelerated approvals of resmetirom and semaglutide in 2024 and 2025, respectively, mark a new era of MASH treatments. I also highlight challenges in MASLD research and speculate on the role of omics and artificial intelligence in our future practice. Finally, I emphasize that the progress today is the result of concerted efforts and years of collaborative work. As long as we work together and embrace change, progress will occur, enabling us to better serve our patients and society.

## 1 Introduction

For younger readers, the *Journal of Gastroenterology and Hepatology Foundation* Okuda State‐of‐the‐Art Lectureship was inaugurated in 2002 to commemorate Professor Kunio Okuda's exceptional contributions to the field. Professor Okuda (1921–2003) was professor and chairman of medicine at the Chiba University School of Medicine [1]. He established the world‐famous liver unit in Chiba and conducted cutting‐edge research on a wide range of hepatology topics, with a special focus on hepatocellular carcinoma (HCC). He served as president of the International Association for the Study of the Liver in 1980, president of the Asian Pacific Association for the Study of the Liver in 1982, and chief editor of the *Journal of Gastroenterology and Hepatology* in 1986.

It is thus with great pleasure and honor that I deliver the Okuda State‐of‐the‐Art Lecture this year as the youngest addition to an incredible list of outstanding lecturers, following my mentor Professor Henry Chan (2018) and my mentor's mentor, Professor Anna Lok (2010). I have had personal interactions with almost all the previous lecturers and consider them my teachers (and friends, if I may say so) along the way. One thing that struck me when going through the history of the Lectureship is that none of the previous lectures focused on fatty liver disease (also known as steatotic liver disease). This highlights how the field has evolved over the years, and I am here to share my personal journey in trying to understand this important disease and in improving the lives of those affected by it.

## 2 From Past to Present

Let us go back as far as possible and start with the Chibanian and Gelasian periods (around 750 000 to 40 000 years ago). This was a time when other ancient humans existed. In particular, Neanderthals, also known as *Homo neanderthalensis*, coexisted with us, *Homo sapiens* , for quite some time. Did Neanderthals have fatty liver? Without tissue remains, this is something we will probably never know. However, in an elegant analysis of over 10 000 ancient and present‐day genomes, the *PNPLA3* gene polymorphism that confers an increased risk of fatty liver disease was found in 100% of Neanderthals [2]. With something so extreme, the only logical explanation is that what we call disease genes today were probably once advantageous. We must understand that food abundance and a sedentary lifestyle are very recent events in evolutionary terms. Compared with carbohydrates and proteins, fat also has a higher energy density (9 vs. 4 cal per gram). Therefore, in the face of food scarcity and a cold climate, the ability to store fat in the liver and elsewhere in the body would confer a survival advantage. Such knowledge, while interesting, also reminds us how poorly prepared we humans are to handle overnutrition.

When I graduated from The Chinese University of Hong Kong in 1999, hardly anyone in the Asia‐Pacific region was interested in fatty liver disease (Table 1). The internal medicine textbook published by our medical school did not even have a chapter on fatty liver. When fatty liver was incidentally found on imaging, most clinicians would tell patients that it was nothing to worry about. There were no registered drug treatments, and colleagues outside hepatology often challenged the necessity of diagnosing fatty liver, arguing that lifestyle advice should be given to patients with obesity and other metabolic diseases anyway. During that period, many manuscripts, especially those written by endocrinologists, often began by describing fatty liver as the hepatic manifestation of the metabolic syndrome. What they really meant, I think, was that fatty liver was “just” the hepatic manifestation of the metabolic syndrome—nothing more.

**TABLE 1:** Fatty liver disease in the past and present.

	2000	2025
Nomenclature	Nonalcoholic fatty liver disease	Metabolic dysfunction‐associated fatty liver disease or metabolic dysfunction‐associated steatotic liver disease
Population prevalence	20%–25%	30%–40%
Perceived importance	Benign condition of no clinical significance	A major cause of cirrhosis, hepatic decompensation, and hepatocellular carcinoma
Assessment	Primarily detected by abdominal ultrasonography Liver biopsy as the only means to assess disease severity	Well‐established clinical care pathway based on simple fibrosis score and second‐line tests such as ultrasound elastography and specific blood biomarkers
Pharmacological treatments	No specific treatment	Role of lifestyle intervention well established Resmetirom and semaglutide have received accelerated approval for the treatment of MASH with F2‐F3 fibrosis Several other ongoing Phase 3 studies
Bariatric surgery	Fatty liver was not a consideration	Histological benefits on MASH demonstrated in a randomized controlled trial and numerous observational studies Impact on major adverse liver outcomes demonstrated in longitudinal studies

Abbreviation: MASH, metabolic dysfunction‐associated steatohepatitis.

Even so, knowledge was accumulating during that period. Undeniably, fatty liver was very common in both well‐conducted epidemiological studies and our routine clinical observations. Professor Jian‐Gao Fan and colleagues reported that the age‐ and sex‐adjusted prevalence of fatty liver was 17.3%, with alcohol and metabolic dysfunction contributing 1.9% and 15.4%, respectively [3]. Compared with today's epidemiology, the prevalence rate reported at that time does not seem impressive at all. However, the report was enough to shake the world and became the cover story of the *Journal of Hepatology* in 2005. Meanwhile, our group published the very first paper on fatty liver, showing that 86% of patients had hepatic inflammation and 26% had fibrosis in a retrospective cohort of 42 patients who underwent liver biopsy for the assessment of nonalcoholic fatty liver disease (NAFLD) [4]. Professors Jeanne Clark and Anna Mae Diehl reported a case of cryptogenic cirrhosis likely due to NAFLD [5]. Larger series subsequently confirmed that burnt‐out NAFLD was the most important cause of cryptogenic cirrhosis [6].

Thus, NAFLD appeared to be more important than previously thought. After consulting my mentors Professor Joseph Sung, Francis Chan, and Henry Chan, I decided to pursue a doctoral degree focusing on the pathological characterization and adipokine profiling of NAFLD [7]. This decision marked the beginning of a journey that has lasted throughout my career.

Twenty‐five years later, NAFLD has been renamed metabolic dysfunction‐associated fatty or steatotic liver disease (MAFLD/MASLD), now accompanied by a new set of diagnostic criteria (Table 1) [8]. It has a staggering global prevalence of 38% and is widely recognized as the fastest growing cause of cirrhosis and HCC [9, 10]. Meanwhile, concerted efforts by researchers have enhanced our understanding of the pathophysiology of MASLD and the performance of noninvasive tests for its evaluation [11]. Additionally, two drugs, resmetirom and semaglutide, have received accelerated approval for the treatment of metabolic dysfunction‐associated steatohepatitis (MASH) with F2–F3 fibrosis [12, 13].

## 3 Evolution of Nomenclature and Definitions

A clear definition of the problem is fundamental to any scientific exploration. In clinical medicine, careful disease definition enables researchers to communicate effectively and compare findings. Otherwise, different studies may include different patient populations, leading to confusion and challenges in validating research outcomes. A notable example in the field of hepatology is acute‐on‐chronic liver failure, and recent efforts to harmonize definitions, with the inclusion of representatives from various professional societies, represent a step in the right direction [14].

**FIGURE 1:**
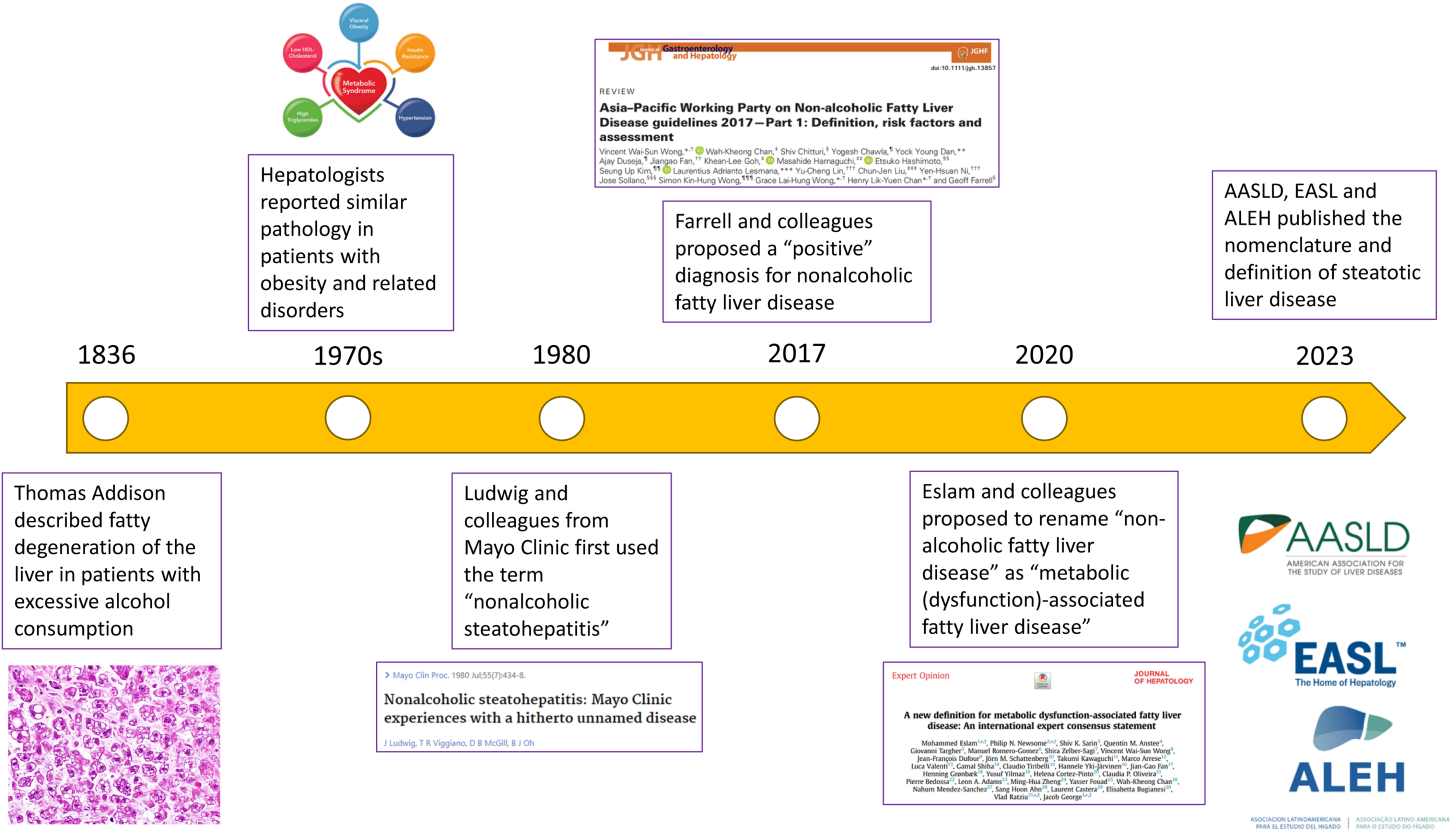
A history of the nomenclature of metabolic dysfunction‐associated fatty and steatotic liver disease.

As for fatty liver disease, Dr. Thomas Addition described fatty degeneration of the liver in patients with excessive alcohol consumption in 1936 (Figure 1) [8]. In the 1970s, hepatologists and pathologists began to recognize that patients with obesity and related disorders could also have fatty liver. In 1980, Dr. Ludwig and colleagues from the Mayo Clinic first described the pathology as nonalcoholic steatohepatitis [15]. Subsequently, NAFLD became the standard term for the disease for the next 40 years. However, there are several shortcomings associated with the name and definition of NAFLD. It is a negative diagnosis based on the presence of fatty liver in patients without excessive alcohol consumption. The term does not explain the drivers of the disease, and the “non” prefix trivializes the condition. In 2017, Professor Geoffrey Farrell proposed a positive definition of NAFLD in the Asia‐Pacific Working Party on NAFLD Guidelines, stating, “NAFLD is a form of fatty liver disease that can reasonably be attributed to overnutrition and its complications, such as weight gain, central obesity, insulin resistance, glucose intolerance, atherogenic dyslipidemia, and arterial hypertension, particularly in genetically predisposed individuals” [16].

To be honest, although I prepared the guidelines with Professor Farrell, I did not understand why he added that section at the time. It was not until 2020, when Professor Mohammed Eslam and Professor Jacob George published the proposal on the new nomenclature and definition of MAFLD, that I fully grasped their meaning [17]. Instead of NAFLD as a diagnosis of exclusion, we can now diagnose MAFLD based on the presence of fatty liver along with cardiometabolic risk factors. MAFLD can also coexist with other liver diseases.

This proposal received mixed responses. The Asian Pacific Association for the Study of the Liver (APASL) and numerous national societies quickly embraced the concept [18], whereas other experts criticized the lack of involvement from stakeholders outside hepatology and the potential harm and confusion that the changes might bring to efforts in biomarker and drug development [19]. As a result, the American Association for the Study of Liver Diseases, the European Association for the Study of the Liver, and the Asociación Latinoamericana para el Estudio del Hígado convened a Delphi process to discuss this issue. In the end, the panel issued a similar proposal but with a few important differences [20]. First, at the suggestion of a patient advocate, the term “steatotic liver disease” was chosen, reasoning that the word “fatty” could be stigmatizing. Second, the cardiometabolic criteria were simplified and aligned with the harmonized criteria of the metabolic syndrome [21]. Because the diagnosis of MASLD only requires the presence of at least one cardiometabolic risk factor, there is almost perfect overlap between the MASLD and NAFLD populations, allowing studies conducted in the NAFLD era to be conveniently extrapolated to MASLD [22]. Third, although MAFLD can coexist with excessive alcohol consumption, steatotic liver disease became the umbrella term under which patients are classified as having MASLD, metabolic dysfunction and alcohol‐associated liver disease (MetALD), or alcohol‐associated liver disease, according to the degree of alcohol consumption.

Several issues deserve mention. The separate publications of the MAFLD and MASLD proposals resulted in some tension among experts and professional societies, which should not be the case. Hepatology has long been a collegial and collaborative specialty. Throughout the history of medicine, there are many examples of a disease having more than one name. For instance, there is no reason why one cannot use “end‐stage kidney disease” and “end‐stage renal disease” interchangeably. Additionally, a subsequent survey suggests that obesity, rather than the term NAFLD, is the main source of stigmatization among patients [23]. In fact, unless we create an environment where people living with various diseases are treated with respect and acceptance, the new term may eventually become stigmatizing. One good example is the evolution of terms like “mental retardation,” “mental handicap,” and “intellectual disability.” Each change was made with good intentions, aimed at reducing stigmatization; however, over time, these labels could still feel stigmatizing and even insulting.

One unexpected consequence of the steatotic liver disease framework has been the significant interest in MetALD among researchers [24, 25]. However, because it was not the original focus, the Delphi panel spent little time considering the nuances of alcohol consumption. In particular, drinking habits often fluctuate over time, making it impractical to change the disease label repeatedly based on short‐term changes [26]. For instance, it would be absurd to say that a patient listed for liver transplantation no longer has alcohol‐associated cirrhosis simply because he has abstained from alcohol for several months. Additionally, the inaccuracy and underreporting of alcohol consumption are well recognized [27], and the role of alcohol biomarkers in research and clinical management requires further clarification [28].

## 4 Noninvasive Assessment: From Diagnosis to Clinical Care Pathway

Although hepatologists rightfully emphasize MASLD as an important cause of cirrhosis and HCC, its epidemiology contrasts starkly with that of other chronic liver diseases and thus deserves a different approach. MASLD affects over one‐third of the global population, with the majority of patients seen in primary care [29]. The large number of MASLD patients with liver‐related complications is primarily driven by this vast population. In absolute terms, the incidence of liver‐related complications from MASLD is lower than that due to other liver diseases, such as chronic viral hepatitis and alcohol‐associated liver disease. Therefore, when recommending tests to identify high‐risk patients, one should consider the cost and availability of these tests, especially outside hepatology settings. It would be ideal to have highly accurate tests that limit unnecessary referrals (i.e., false positives) while avoiding the omission of high‐risk patients (i.e., false negatives).

Among the histological features of MASLD, the degree of liver fibrosis has the strongest correlation with progression to cirrhosis, hepatic decompensation, the need for liver transplantation, and liver‐related death [30]. Consequently, noninvasive tests for fibrosis have been the focus of research in biomarker discovery and validation. Noninvasive tests for fibrosis can generally be classified into simple fibrosis scores, specific blood biomarkers, and imaging biomarkers.

Simple fibrosis scores are typically derived using histological fibrosis stage as the reference standard. Among them, the Fibrosis‐4 index (FIB‐4), NAFLD fibrosis score, and the aspartate aminotransferase (AST)‐to‐platelet ratio index are the most extensively evaluated [11]. In particular, FIB‐4 can be calculated using age, AST, alanine aminotransferase (ALT), and platelet count. These are relatively inexpensive blood tests that can be performed in primary care settings. At a cutoff of 1.3 (or 2.0 in patients older than 65 years), FIB‐4 has a high negative predictive value in excluding F3‐F4 fibrosis.

Specific blood biomarkers measure fibrogenesis and/or fibrinolysis. The more extensively evaluated biomarkers include the enhanced liver fibrosis score (ELF), PRO‐C3, and the PRO‐C3‐based ADAPT score [11, 31]. Some of these biomarkers have been included in clinical trials of MASH as secondary or exploratory endpoints. Although the availability of these biomarkers remains limited, one potential advantage is that they can be ordered by primary care physicians without the need for specialized equipment. Furthermore, with the right arrangement, specific blood biomarkers can be performed as a reflex test in cases of abnormal FIB‐4, thus simplifying the diagnostic process and improving efficiency.

Vibration‐controlled transient elastography (VCTE) is commonly performed at tertiary centers in the Asia‐Pacific region and Europe, although other imaging biomarkers, such as point‐shear wave elastography, two‐dimensional shear wave elastography, magnetic resonance elastography (MRE), and corrected T1, are also increasingly used [32]. Both ultrasound‐ and magnetic resonance imaging‐based techniques allow for assessment of both liver fibrosis and steatosis, providing useful information for patients with MASLD.

**FIGURE 2:**
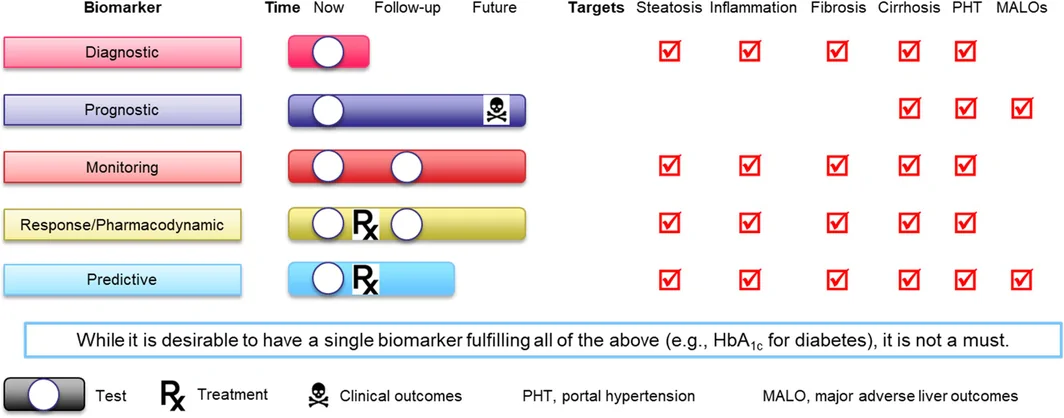
Application of biomarkers in MASLD.

Although most biomarker studies focus on the diagnosis of fibrosis, it is important to note that biomarkers can also be used for other purposes, including prognostication, monitoring disease progression, and assessing treatment response (Figure 2). Patients with persistently low FIB‐4 have a low incidence of liver‐related complications [33, 34, 35]. In the multicenter VCTE‐Prognosis study, baseline Agile scores (composite scores based on liver stiffness measurement by VCTE, age, sex, diabetes, AST, ALT, and platelet count) demonstrated superior performance in predicting hepatic decompensation and HCC compared with VCTE alone, liver histology, and other simple fibrosis scores [36]. Importantly, the study demonstrated a correlation between changes in noninvasive tests and corresponding changes in the risk of liver‐related complications.

In head‐to‐head comparisons, MRE is marginally superior to VCTE in diagnosing mild and moderate fibrosis and has a higher measurement success rate [37, 38]. Not surprisingly, MRE and its derived MEFIB and MAST scores also correlate with clinical outcomes [39], but data on their monitoring role with serial measurements remain scarce [40].

Based on the existing evidence, professional societies have issued largely consistent recommendations on the clinical care pathway for MASLD, using simple fibrosis scores such as FIB‐4 for initial assessment, followed by a second‐line test like VCTE or ELF in cases of abnormal FIB‐4 [41, 42, 43, 44]. Using the VCTE‐Prognosis cohort, we demonstrated that this two‐step approach is also prognostic [45]. Importantly, the performance of this two‐step approach in predicting hepatic decompensation and HCC was almost equivalent to performing VCTE on all patients with MASLD, suggesting that the former is more cost‐effective.

Although there is a wealth of literature on the application of noninvasive tests and clinical care pathways, implementing such knowledge in routine clinical practice remains a challenge, especially in primary care and other specialties where MASLD is seldom a priority. In my opinion, in low‐awareness settings, this clinical care pathway can only be implemented if it becomes part of standard or automated practice. Several studies have confirmed the feasibility of performing VCTE in diabetes units alongside assessments of other diabetic complications [46, 47, 48]. In collaboration with Professor Wah‐Kheong Chan, our group conducted the multicenter NAFLD‐NIT‐RCT and showed that automated fibrosis score calculations, followed by flagging abnormal test results, increased referrals of patients with abnormal fibrosis scores by tenfold [49].

Despite the progress outlined above, questions remain. FIB‐4 is imperfect and would miss a significant proportion of patients with advanced liver disease, especially in high‐risk populations such as those with type 2 diabetes [50]. Some experts have suggested the possibility of lowering the cutoff for FIB‐4 to increase its sensitivity. However, based on our findings in the NAFLD‐NIT‐RCT, this is unlikely to be effective. Using the current cutoffs, 30% of patients with type 2 diabetes already required a second‐line test, among whom only 20% had liver stiffness measurements above 10 kPa [49]. Lowering the FIB‐4 cutoff would result in even more patients being referred, thus increasing the false‐positive rate.

Therefore, several groups have derived and validated improved fibrosis scores (e.g., SAFE score, MAFLD fibrosis score, LiverRisk score, LiverPRO score, and MAF‐5 score) (Table 2) [51, 52, 53, 54, 55]. Some of these studies used traditional approaches, employing liver histology as the reference standard. In contrast, the LiverRisk score and MAF‐5 score were modeled against VCTE in community settings, making them more applicable in primary care [53, 55]. All of these scores have undergone external validation and demonstrated superiority over FIB‐4. Nonetheless, more data are needed, especially regarding the role of monitoring with serial measurements.

**TABLE 2:** New liver fibrosis scores underdevelopment.

Score	Components	Derivation population	Target condition
SAFE score	Age, BMI, DM, PLT, AST, ALT, and globulins	NASH CRN	F2‐F4 fibrosis
MAFLD fibrosis score	Age, BMI, INR, AST, GGT, PLT, and DM	MASLD biopsy cohort	F3‐F4 fibrosis
LiverRisk score	Age, sex, glucose, cholesterol, AST, ALT, GGT, and PLT	VCTE in general population	VCTE‐LSM 6, 10, and 15 kPa
LiverPRO score	Age + 3–9 blood tests	SLD biopsy cohort	F2‐F4 fibrosis
MAF‐5 score	Waist circumference, BMI, AST, PLT, and DM	NHANES 2017‐2020	VCTE‐LSM 8 kPa

Abbreviations: ALT, alanine aminotransferase; AST, aspartate aminotransferase; BMI, body mass index; DM, diabetes mellites; GGT, gamma‐glutamyl transpeptidase; INR, international normalized ratio; LSM, liver stiffness measurement; MAFLD, metabolic dysfunction‐associated fatty liver disease; MASLD, metabolic dysfunction‐associated steatotic liver disease; NASH, nonalcoholic steatohepatitis; PLT, platelet count; SLD, steatotic liver disease; VCTE, vibration‐controlled transient elastography.

## 5 A New Era of Mash Therapies

Lifestyle intervention through a healthy diet and regular exercise remains the cornerstone of managing MASLD. Studies have shown a dose–response relationship between the degree of weight reduction and improvements in MASLD [56, 57]. It is often stated that a 5%–7% weight reduction is required for MASH resolution, whereas over 10% is necessary for fibrosis improvement. However, there is considerable individual variability; some patients show improvement with a lesser degree of weight reduction [58]. Despite this, even in clinical trial settings, only 10%–20% of patients manage to lose more than 10% of their body weight, and even fewer can maintain this weight reduction over time. Thus, it remains crucial to develop therapies for MASH.

For a long time, MASH was considered a bleak field. In the early 2000s, there was limited commercial interest, and researchers could only explore existing agents such as vitamin E, thiazolidinediones, and metformin [59]. By the late 2010s, the MASH field became more active but was plagued by a series of failed clinical trials. The spectacular failures of large Phase 3 programs involving selonsertib, cenicriviroc, and elafibranor raised doubts about the achievability of regulatory requirements [60, 61]. Nevertheless, the field learned valuable lessons (Table 3). Above all, pure anti‐inflammatory or antifibrotic drugs do not significantly advance treatment. This should not surprise us; years of hepatology research have shown that addressing the underlying etiology is the most effective means to reverse liver fibrosis and prevent cirrhosis and its complications. If we find it illogical to treat a patient with chronic viral hepatitis using an anti‐inflammatory drug instead of antivirals, we must question how we could treat MASH without addressing its primary metabolic drivers. Second, no matter how eager we are for positive results, we must be honest and avoid overinterpreting isolated positive endpoints that are not corroborated by other biomarkers. For instance, although cenicriviroc appeared to increase the rate of fibrosis improvement at 1 year in the Phase 2 CENTAUR study, this beneficial effect was not accompanied by improvements in inflammation or noninvasive tests of fibrosis [62]. This alone should have raised doubts about whether the observed fibrosis improvement was a chance finding. Eventually, no fibrosis improvement was noted in the subsequent Year 2 results of the CENTAUR study or in the Year 1 interim analysis of the Phase 3 AURORA study [61, 63].

**TABLE 3:** Lessons learned from failed MASH trials.

Pure anti‐inflammatory or antifibrotic drugs do not work in MASH if the metabolic dysfunction is not controlled.
Current in vitro and in vivo models do not fully recapitulate the pathophysiology of MASH, so there can be a disconnect between promising preclinical data and subsequent clinical trials.
A really effective drug would not just improve a single endpoint. If a positive endpoint is not supported by concomitant changes in other related and unrelated biomarkers, the result should be interpreted with caution.
Biomarkers should be interpreted in the right context. Even for an effective treatment, some biomarkers (e.g., for steatosis) may improve much earlier than others (e.g., for fibrosis). Furthermore, some biomarkers may be more suitable for drugs with particular modes of action.
Inadequately powered early phase clinical trials often yield misleading results.
Findings in a precirrhotic cohort cannot be extrapolated to the cirrhotic population.
Above all, do no harm. A positive liver‐centric response cannot offset harmful effects, especially in regard to cardiovascular risk in the setting of MASH.

Abbreviation: MASH, metabolic dysfunction‐associated steatohepatitis.

Another example is obeticholic acid, a farnesoid X receptor agonist, which demonstrated antifibrotic effects in the Phase 3 REGENERATE study but ultimately did not reach the market [64]. This time, the issues were related to tolerability and safety: approximately half of the patients receiving high‐dose obeticholic acid experienced pruritus, and the drug led to atherogenic dyslipidemia. Given that cardiovascular disease remains the leading cause of death in MASH, the safety of therapeutic agents cannot be overstated.

Despite these setbacks, the industry and researchers persevered. On March 14, 2024, resmetirom, a liver‐specific thyroid hormone receptor‐beta agonist, became the first drug to receive accelerated approval for treating MASH with F2‐F3 fibrosis. This decision was based on the Phase 3 MAESTRO‐NASH study, which demonstrated both MASH resolution and fibrosis improvement at the week 52 interim analysis [12]. Reflecting the lessons mentioned above, resmetirom not only improved liver histology but also a wide range of secondary endpoints. Since early phase studies, the cardiovascular safety of resmetirom has been meticulously evaluated. The late Professor Stephen Harrison devoted his career to MASH therapies and would likely be very pleased with this historic achievement.

The most significant breakthrough in managing obesity and metabolic disorders over the past decade has come from the discovery of glucagon‐like peptide‐1 (GLP‐1) and the development of GLP‐1 receptor agonists, which led to the 2024 Lasker–DeBakey Clinical Medical Research Award being presented to Drs. Svetlana Mojsov, Joel Habener, and Lotte Bjerre Knudsen [65]. GLP‐1 receptor agonists have profound effects on appetite suppression and metabolism. They improve glycemic control and body weight and have positively impacted numerous obesity‐related outcomes, including major adverse cardiovascular events, heart failure with preserved ejection fraction, obstructive sleep apnea, and musculoskeletal disorders. Professor Philip Newsome's team pioneered this field, first demonstrating the beneficial effects of liraglutide on MASH histology in the LEAN study [66]. The positive week 72 interim analysis of the ESSENCE study led to the accelerated approval of semaglutide in August 2025 [13]. However, this is just the beginning. New incretin therapies with dual or triple agonism targeting GLP‐1, glucagon, and glucose‐dependent insulinotropic polypeptide receptors promise to achieve even greater weight reduction and potentially more profound effects on MASH [67, 68]. Retatrutide, a triple agonist, demonstrated over an 80% relative reduction in liver fat fraction and normalization of liver fat in about 90% of treated patients in a 48‐week Phase 2 study [69].

Another breakthrough in MASH therapeutics is the development of fibroblast growth factor 21 (FGF21) analogues. FGF21 is recognized as a master metabolic molecule. Remarkably, three different FGF21 analogues (efruxifermin, pegozafermin, and efimosfermin alfa) all demonstrated potent antifibrotic effects in Phase 2 studies, suggesting genuine benefits [70, 71, 72]. Notably, 75% of patients receiving efruxifermin 50 mg weekly achieved fibrosis improvement without worsening MASH by week 96. In another Phase 2 study involving patients with compensated MASH cirrhosis, fibrosis improvement without worsening MASH at week 96 was observed in 11% of the placebo group, 21% of the 28‐mg efruxifermin group, and 29% of the 50‐mg efruxifermin group, making efruxifermin the first drug to demonstrate the reversal of MASH cirrhosis [73].

Other drugs underdevelopment include the pan‐peroxisome proliferator‐activated receptor agonist lanifibranor and inhibitors of de novo lipogenesis [74, 75, 76]. With accumulating knowledge of the genetic polymorphisms associated with MASLD, short interfering RNA and antisense oligonucleotides have been developed to inhibit the expression of MASH genes, with *PNPLA3* and *HSD17B13* being the most advanced in development.

## 6 Future Perspectives

**FIGURE 3:**
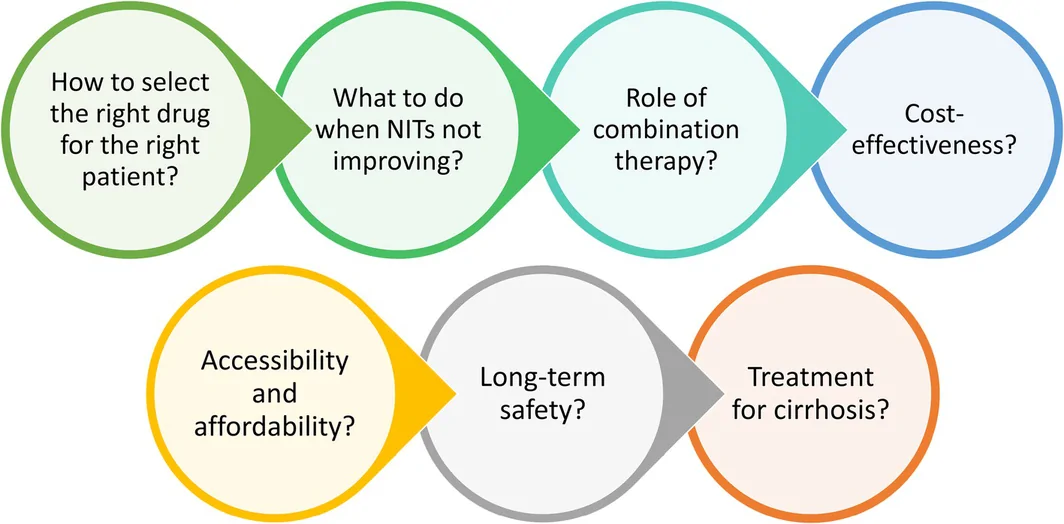
Considerations after initial approvement of MASH drugs. NIT, noninvasive test.

The accelerated approval of the first two drugs for MASH treatment is only the beginning. Much still needs to be done to determine how best to select the right drug for the right patient (Figure 3). We must accurately define response and nonresponse using noninvasive tests in real‐world settings. Ongoing trials will also assess whether the observed histological improvements will ultimately translate into enhanced clinical outcomes with long‐term safety. Equally important is ensuring that these drugs are accessible and affordable for patients in need.

The explosion of cutting‐edge omics technologies promises to bring precision medicine closer to reality. Although many new technologies may currently seem distant from clinical practice, advancements and subsequent price reductions—similar to those seen in whole genome sequencing over the past two decades—could break down existing barriers. In fact, direct‐to‐consumer genomic sequencing services are already available in some countries, and it is not far‐fetched to envision a future where patients have had their genomes sequenced. Clinicians must prepare themselves to handle such information.

There is no shortage of conflicting studies regarding whether MASLD is associated with specific conditions. One possible explanation is that our understanding of MASLD has been too simplistic. Hepatic steatosis is a biological phenomenon resulting from numerous factors, including genetics, lifestyle, and cardiometabolic comorbidities, with the relative importance of each factor varying among individuals. Recent clustering analyses suggest that different subtypes of MASLD have varying risks of cardiometabolic and liver‐related outcomes [77, 78]. Therefore, understanding the dominant pathophysiology in a particular patient will enable us to provide more targeted and effective therapies and determine whether a patient requires a liver‐targeted therapy at all.

A discussion of the future would not be complete without mentioning artificial intelligence (AI). In our field, AI has already significantly contributed to the assessment of imaging and histology, as well as the development of various prediction models [79, 80, 81]. Recently, Dr. Shmatko and colleagues reported using generative transformers to create an AI tool capable of predicting more than 1000 diseases with accuracies comparable to or surpassing those of models previously developed for individual diseases [82].

Bill Gates once said, “Most people overestimate what they can do in one year and underestimate what they can do in ten years.” Our predictions for the future are often incorrect. Unlike what I read in science fiction during my secondary school years, we still do not have flying cars, and humans have yet to establish a presence on the Moon or Mars. However, we do have many advancements. For instance, the computing power and functionality of something as simple as the smartphone in our pockets far exceed the wildest dreams of sci‐fi writers. Our inability to predict the future should not deter us from dreaming. As long as we work together and embrace change, progress will occur, enabling us to better serve our patients and society.

## Funding

Dr. Wong receives research funding from the Research Grants Council of the Hong Kong SAR Government (General Research Fund project number 14106923 and 14106824), Noncommunicable Chronic Diseases‐National Science and Technology Major Project of China (project number 2023ZD0508700), and The Chinese University of Hong Kong (project number 2025.034).

## Conflicts of Interest

Dr. Wong served as a consultant or advisory board member for AbbVie, AstraZeneca, Boehringer Ingelheim, Echosens, Eli Lilly, Gilead Sciences, Merck, Novo Nordisk, Pfizer, and TARGET PharmaSolutions; and a speaker for Abbott, AbbVie, Echosens, Gilead Sciences, and Novo Nordisk. He has received a research grant from Gilead Sciences and is a co‐founder of Illuminatio Medical Technology.

## Data Availability

Research data are not shared.
